# Does Propolis Help to Maintain Oral Health?

**DOI:** 10.1155/2013/351062

**Published:** 2013-01-09

**Authors:** Włodzimierz Więckiewicz, Marta Miernik, Mieszko Więckiewicz, Tadeusz Morawiec

**Affiliations:** ^1^Department of Prosthetic Dentistry, Faculty of Dentistry, Wrocław Medical University, 50425 Wrocław, Poland; ^2^Division of Dental Materials, Faculty of Dentistry, Wrocław Medical University, ul. Krakowska 26, 50425 Wrocław, Poland; ^3^Department of Oral Surgery, Faculty of Medicine and Dentistry, Medical University of Silesia in Katowice, 41902 Bytom, Poland

## Abstract

Propolis, known also as bee glue, is a wax-cum-resin substance which is created out of a mix of buds from some trees with the substance secreted from bee's glands. Its diverse chemical content is responsible for its many precious salubrious properties. It was used in medicine already in ancient Egypt. Its multiple applications during the centuries have been studied and described in details. The purpose of this study is to present the possible use of propolis in treatment of various diseases of oral cavity in their dental aspect. The paper presents properties and possible applications of bee glue depending on dental specialities. An overview of publications which appeared during the recent years will allow the reader to follow all the possibilities to apply propolis in contemporary dentistry.

## 1. Introduction

Propolis is a wax-cum-resin substance that is produced by bees. The word itself comes from ancient Greek, means an outer wall of a city (pro: before, polis: city) and relates to the protective properties of the substance. Bees use it to protect and reinforce their hives, repair their structure, and to cover honeycombs. It kills pathogens, protects against rain and being a very sticky substance, prevents unwanted guests from entering the hive [1–3]. Not all species of bees produce bee glue at the same degree [4]. The colonies of Apis dorsata, called giant honey bee, use propolis to strengthen adhesion of the hive, while Apis cerana does not use it at all. Apis mellifera is the species which uses propolis in every possible way [5]. Bee glue is made from substances collected by bees from tree buds which are then digested and mixed with the substance secreted by bee's glands. It is dark green or brown and its chemical content depends on the geographic zone from which it comes [6]. Most often propolis is composed of: resins (40–55%), bee wax and fatty acids (20–35%), aromatic oils (about 10%), pollen (about 5%), and other components like minerals and vitamins. Nevertheless, their presence and percentage content in propolis changes and depends on their origin, the type of plant pollen, and the species of bees that produced it [7, 8]. The results of the study published by Dias et al. present the percentage content of phenol compounds within the range between 11.1 ± 1.3% and 28 ± 2.0%, flavonoids between 3.1 ± 0.3% and 12.0 ± 0.3% for propolis from different regions of Portugal [9]. Choi et al. defined the range of phenol compounds between 12.0% and 21.2% for propolis from different areas of Korea [10]. The research carried out by Inouye et al. showed that one of varieties of Japanese propolis contains neither flavonoid nor phenolic acid [11]. The composition of chemical compounds is responsible for the properties of propolis. Application of bee adhesive in medicine has been described extensively. It has antibacterial, antifungal, anti-inflammatory, anticancer, antiviral, immunostimulator, and many other properties [12–21]. A wide spectrum of its reaction allows to use it in many medical specialisations. Contemporary dentistry is an inseparable part of medicine and therefore attempts were made to use propolis in dentistry, as well. 

The aim of this paper is to present the possibilities to apply propolis in various branches of dentistry on the basis of chosen articles available from PubMed, PubMed Central, and CINAHL databases that were published between 1976 and 2012. The paper which have been selected include valuable original articles and case reports related to terms: propolis, dentistry, bee glue, and allergy.

## 2. Use of Propolis in Dental Specialties

### 2.1. Oral Hygiene

Mouth environment is rich in bacterial flora which in some conditions may lead to such diseases like caries or diseases of periodontium [22, 23]. 

The basic role in development of dental caries plays Streptococcus mutans and, to a lower degree, Lactobacillus sp.. Cariogenic influence of other bacteria including Streptococcus, Enterococcus, or Actinomyces is disputable [24]. Virulence of Streptococcus mutans results from its ability to adhesion, acid-forming properties, and tolerance to environment with low pH [25]. In order to prevent dental caries an attempt was made to analyse the influence of propolis on mouth environment and bacterial flora, in particular on S. mutans. In 1991, Ikeno et al. proved that propolis considerably reduces teeth caries in rats as the result of its multidirectional influence on bacterial flora: it limits the number of microorganisms, slows down synthesis of insoluble glucans, and slows down activity of glucosyltransferase [26]. Studies done by other authors unanimously show that extracts from bee glue limit the quantity of bacterial plaque which influences the reduction of tooth caries [27–34]. Duarte et al. explained cariostatic effects of propolis by high quantity of fatty acids which slow down the production of acids by Streptococcus mutans and decreases the tolerance of microorganisms to acid pH [35]. Özan et al. and Arslan et al. proved that propolis-based solutions are not as effective as chlorhexidine gluconate solutions in prevention of caries; nevertheless, their anticaries impact was statistically important in comparison with a control group [36, 37]. The study done by Özan et al. shows, however, that propolis-based solutions have lower cytotoxic effect on the cells of human gum fibroblasts than chlorhexidine, which predisposes them to be used as ingredient of mouthwashes [36]. Nevertheless, the research done by Murray indicated that the effect of propolis extract on reducing bacterial plaque growth is marginal [38]. In this case, the effect of use of propolis was slightly better than in the case of a control group, however, statistically it was negligible. In most researches propolis is used directly in the mouth in the form of ethanol- or water-based mouth rinses [27, 28, 31, 32, 34–36, 38] or in the form of a toothpaste [29, 30]. Propolis can be also used in a form of a solution to decontaminate fibres of toothbrushes [39]. 

Bacterial flora of the mouth can cause not only caries but also periodontal diseases. Bacterial plaque accumulated over and under gums contributes to inflammation of the tissues adjacent to teeth which leads to clinical attachment loss and a loss of alveolar process [40, 41]. Socransky et al. divided microbes which are located in the subgingival plaque into five complexes. One of them, the “red complex,” made up of Tannerella forsythensis, Porphyromonas gingivalis, and Treponema denticola has strong relation with an increased depth of periodontal pockets and with a bleeding on probing [42]. Results of some studies indicated also Prevotella intermedia and Fusobacterium nucleatum as potential etiologic factors of periodontitis [43]. A decrease of the number of these pathogenic microbes could potentially influence epidemiology of periodontal diseases by a limitation of their number and intensity. A research by Koo et al. indicated high effectiveness of a propolis extract on reducing growth of bacteria that belong to red complex [28]. Also Santos et al., Feres et al., and Koru et al. confirmed antibacterial properties of propolis in relation to pathogens of periodontitis [44–46]. Santos et al. indicated also that antibacterial effects are conditioned by flavonoids, phenol acids, and their esters [44]. A research carried out by Tanasiewicz et al. showed clinical effectiveness of a toothpaste and gel containing 3% ethanolic extract of propolis in a group of patients with a greater risk of gingivitis caused by dental plaque [47]. As propolis mouth rinses and propolis-based toothpastes stop the growth of pathogens of gingivitis and periodontitis, they seem to be promising not only as preventive but also as therapeutic agents [30, 45, 48, 49]. The results of the study by Sonmez et al. showed, however, that propolis extracts in concentration that effectively reduces pathogenic organisms for periodontal diseases are cytotoxic for the gingival fibroblasts [49]. Preventive effect of propolis on periodontal tissues includes also the slowing down of formation of precipitates of calcium phosphates and because of that, it can be used as ingredient of mouthwashes or toothpastes in order to limit the accumulation of dental plaque [50]. 

Halitosis, an unpleasant breath, is also largely related to hygiene of the oral cavity. The byproducts of degradation of microorganisms located in the mouth are one of the reasons of bad breath [51]. Microbes particularly related to the creation of bad breath include the red complex bacteria and: Prevotella intermedia, Porphyromonas endodontalis, and Eubacterium [52]. The measurements of the content of volatile sulfur components in exhaled air with the use of Halimeter done by Sterer and Rubinstein [53] and Barak and Katz [54] indicate that propolis reduces halitosis. Nevertheless, propolis is not as effective as zinc-, echinacea- or lavender-based products.

### 2.2. Periodontology and Oral Mucosa Pathologies

The confirmation of effectiveness of propolis in fighting etiological factors of periodontitis made some authors include these preparations in the periodontologic therapeutic protocol. Bruschi et al. proved that mucoadhesive hydrophilic gel that contains propolis, when applied to gingival pockets, can be useful in treatment of periodontitis [55]. Research done by Coutinho allowed to conclude that additional subgingival irrigations with a propolis extract during periodontologic treatment allowed to obtain better results than scaling and root planning by themselves, which results from the assessment of both clinical and microbiological parameters [56]. For this reason, it should be considered to include this type of therapy in the algorithm of periodontitis treatment. Not only local, but also oral use of propolis-based preparations turns out to be effective in periodontal treatment. Toker et al. carried out a study which on the basis of a morphologic and histologic picture showed that oral application of propolis prevents the loss of alveolar process bone in the case of periodontitis in rats [57]. 

Herpes simplex, the virus which causes a disease of mouth mucosa, is one of most popular human pathogens [58]. In cases of infection caused by this virus attempts were made to use propolis-based extracts in its treatment. The use of propolis solutions by Schnitzler et al. disclosed that bee glue has high antiviral effectiveness. It was also found that single components of propolis do not have the same antiviral effect as their mixture in the form of bee glue. This is the basis for the conclusion that propolis extracts can be used locally in viral infections [59]. The research done by Shimizu et al. indicates that propolis delays growth and progression of skin changes in an early stage of infection with Herpes simplex and does not cause cytotoxic effect [60]. Propolis is also used in treatment of recurrent aphthoid stomatitis. Although it is a common disease whose symptoms appear in the mouth, its exact etiology has not been found yet which makes the therapy more difficult [61]. Bee glue turned out to be effective in the treatment as it lowers down the frequency of recurrence of the disease and improves the quality of life of patients who suffer from recurrent stomatitis [62]. Bee glue-based preparations seem also to be useful in treatment of stomatitis caused by chemotherapy, however, more research has to be done on this subject [63]. 

### 2.3. Oral Surgery

In dental surgery, propolis is used in replantation of avulsed permanent teeth and supports the healing process after surgery in the oral cavity. 

Maintenance of alive periodontal cells is one of the crucial factors that condition a successful replantation of an avulsed permanent tooth. For this reason, many studies were conducted in order to find the best means to transport the complete displacement teeth [76]. Most scientific studies show very good effects of storage of avulsed teeth in propolis. In the study done by Özan et al. propolis turned out to be a better means for transportation than milk or Hank's Balanced Salt Solution [65]. Also research done by Mori et al. in rats and laboratory study done by Saxena et al. recommend propolis as their choice for a means of transport [64, 66]. Gulinelli et al. state that there are no statistically important differences between storage of a avulsed tooth in physiological saline, sodium fluoride, or propolis in relation to the effects of a delayed replantation of a tooth [77]. Bee glue seems, however, to be less effective in comparison with coconut milk which allows maintaining a bigger quantity of alive cells of periodontium [78]. Nevertheless, a recent study done by Gjersten et al. indicates that propolis is extremely effective; it not only reduces apoptosis of periodontium cells but also increases their metabolic activity and proliferation [79]. 

Magro-Filho and de Carvalho proved that local application of propolis helps to heal wounds after a surgery within the oral cavity, reduces inflammation and has analgesic effect [80]. Also Lopes-Rocha et al. noted a beneficial effect of bee glue on healing of surgical wounds within the oral cavity. Propolis decreases inflammation and speeds up creation of granulation tissue and epithelialization [67].

### 2.4. Orthodontics

In malocclusions accompanied by a considerable narrowing of the maxilla, it is necessary to use a device to expand the palatine suture. During the treatment bone remodeling takes place within the palatine suture. The research carried out by Altan et al. on rats confirms positive effect of propolis solution on bone forming process during the treatment with the device to expand the palatine suture [68]. The results of the research show an increased quantity of osteoblasts in preparations from rats which received propolis during the treatment. In such cases the bone remodeling within the palatine suture was quicker. 

### 2.5. Restorative Dentistry

In restorative dentistry, propolis can be used to decrease permeability of the dentin and to direct pulp capping in order to create restorative dentin. 

 Sales-Peres et al. found that propolis can reduce dentin permeability. On this basis, it can be concluded that it counteracts tooth sensitivity. This feature results from the fact that bee glue has the capacity to partially impregnate dental tubules [70]. 

The direct pulp capping after mechanical or chemical uncovering is made in order to stimulate the pulp to create restorative dentin. The regenerative effect of propolis on the tooth pulp has been known for a long time [81]. Nevertheless, there is no consent on the subject of propolis extracts effectiveness in comparison with calcium hydroxide which is most often used in stimulation of creation a reparative dentin. Bretz et al. state that there are no important differences in direct capping with propolis and with calcium hydroxide-based products. Both of them offer a similar degree of healing pulp inflammation, reducing quantity of microbes and stimulating creation of dentin bridge [82]. Also the studies carried out by Parolia et al. and Ozório et al. indicate that propolis, calcium hydroxide, and MTA have similar effectiveness in induction to create reparative dentin [69, 83]. However, the results of the research done by Ahangari et al. prove clearly higher effectiveness of direct pulp capping with propolis than with calcium hydroxide-based products. It not only stops inflammatory reaction, infection with microbes and pulp necrosis but also induces formation of high quality tubular dentin through stimulation of stem cells [84]. According to Sabir et al., the simulative effect on dental pulp is conditioned by presence of flavonoids in propolis extracts [85]. 

### 2.6. Endodontics

One of the aims of endodontic treatment is a complete elimination of microbes in root canals [86]. The effectiveness of medicines used in endodontology is often assessed through a test of *Enterococcus faecalis* growth which is resistant to unfavorable environment and can survive in root canal system despite application of some medicaments [87]. Many studies show that propolis effectively limits the quantity of *E. faecalis* in root canals [71, 72, 88–91]. The studies done by Kayaoglu et al. and Mattigati et al. indicated that effectiveness of propolis in decontamination of root canals is lower than that of chlorhexidine [71, 72]. Some researches indicate that propolis is more effective in fighting microbes than calcium hydroxide-based products [71, 72]. Some authors proved that bee glue has antibacterial properties similar to calcium hydroxide or worse [92]. Such differences may result from different times of measurement. Cuevas-Guajardo et al. carried a research which show that calcium hydroxide is more effective than propolis up to 24 hours from application. After 48 and 72 hours a mix of propolis with calcium hydroxide with volume proportion 1 : 3 showed best antibacterial properties [93]. Because of inconsiderable inflammation of periapical tissue and protective effect on the cells of periodontium, propolis can be effectively used as a product to disinfect the root canals [73, 94]. 

### 2.7. Prosthetic Dentistry

Denture stomatitis is a frequent pathology in patients who use removable dentures. Etiological factors of this disease include, first of all, an infection with *Candida albicans*, an incorrect hygiene of the oral cavity and prolonged use of dental prosthesis [95]. Propolis-based products have strong antifungal properties in relation to *Candida albicans* and to other types of Candida sp, whereas *Candida albicans* is most sensitive to propolis [28, 32, 96, 97]. Propolis solutions can be used in form of mouthwash [74, 97] or a gel for local application [75] in therapy of patients with oral candidiasis connected with use of removable dentures. However, da Silva et al. proved that a gel which contains propolis, used in treatment of denture stomatitis, can have a negative effect on the structure of the surface of acrylic resin which becomes rough and more prone to adhesion of microbes [98]. 

## 3. Discussion

In spite of many benefits and possibilities of application of propolis in dentistry, presented in Table 1, there is a risk of allergy to it. The results of the study by Münstedt and Kalder in a group of 41 German bee beekeepers indicate that 70.7% of them had the symptoms of a contact allergy after 9.5 years of professional work, on average [99]. Its symptoms were mostly limited to itching and a rash. The allergy for the second group was confirmed by skin tests. Brailo et al. described a case of a generally healthy 20-year-old patient who had irregular erosions partially covered with pseudomembranes that involved both lips and retrocomissural mucosa [100]. She stated that she had used a propolis solution in treatment of aphthous ulcers. The changes in the mucosa appeared after 10 days of using the product. A late contact allergy to propolis was diagnosed. She was instructed to discontinue the use of propolis-based product and to use 0.05% betamethasone 3 times a day for 14 days. The patient followed the instructions and the changes on lips and the mucosa began to disappear. Zirwas and Otto state that during the last 10 years allergy to propolis has increased from 0.5% to 1.4% [101]. This can be an important discovery, considering that bee glue is more and more often used as a component of toothpastes, chewing gums, creams, and ointments. For this reason, the research on propolis has to be continued in order to define the algorithms of its application in various branches of dentistry on the basis of its biological activity. 

## 4. Conclusions

Propolis-based preparations have a wide range of applications in various specialities of dentistry. Thanks to the richness of natural components in it, bee glue has antibacterial, antiviral, antifungal, anti-inflammatory, analgesic, and many other applications. Although it offers many benefits, one should remember that its use may bring in the risk of an allergy.

## Figures and Tables

**Table 1:** Selected propolis activities according to dental specialties.

Dental specialities	Propolis activity	Type of studies	Year of publication	Authors
	(i) Component of toothpastes	Human	2001	Botushanov et al. [30]
	(ii) Component of daily mouthwash	Human	2007	Özan et al. [36]
Oral hygiene	(iii) Antibacterial (toothbrushes decontamination)	Laboratory	2012	Bertolini et al. [39]
	(iv) Component of preventive toothpaste and gel against gingivitis	Human	2012	Tanasiewicz et al. [47]

	(i) Antibacterial	Laboratory	2002	Santos et al. [44]
	(ii) Mucoadhesive gel against periodontitis	Laboratory	2007	Bruschi et al. [55]
	(iii) Prevents alveolar bone loss	Animal	2008	Toker et al. [57]
Periodontology and oral mucosa pathologies	(iv) Regeneration of periodontal ligaments	Laboratory	2011	Saxena et al. [64]
	(v) Mouthwash against gingivitis	Human	2011	Pereira et al. [48]
	(vi) Antiviral	Animal	2011	Shimizu et al. [60]

Oral surgery	(i) Storage media for avulsed teeth	Laboratory and Animal	2007 and 2010	Özan et al. and Mori et al. [65, 66]
	(ii) Healing of oral surgical wounds	Animal	2012	Lopes-Rocha et al. [67]

Orthodontics	(i) Hasten a new bone formation at the expanded midpalatal suture	Animal	2012	Altan et al. [68]

Restorative Dentistry	(i) Direct pulp capping	Human	2010	Parolia et al. [69]
	(ii) Treatment of dentin hypersensitivity	Laboratory	2011	Sales-Peres et al. [70]

Endodontics	(i) Antibacterial against root canals bacteria	Laboratory	2011 and 2012	Kayaoglu et al. and Mattigati et al. [71, 72]
	(ii) Temporary root canal filling after pulpectomy	Animal	2012	Ramos et al. [73]

Prosthetic dentistry	(i) Antifungal	Human	2005	Santos et al. [74]
	(ii) Treatment of denture stomatitis	Human	2008	Santos et al. [75]
